# Impact of sterilization and chemical fertilizer on the microbiota of oil palm seedlings

**DOI:** 10.3389/fmicb.2023.1091755

**Published:** 2023-04-27

**Authors:** Joyce Yoon Mei Ding, Li Sim Ho, Julia Ibrahim, Chee Keng Teh, Kian Mau Goh

**Affiliations:** ^1^Biotechnology & Breeding Department, Sime Darby Plantation Technology Centre Sdn. Bhd., Serdang, Selangor, Malaysia; ^2^Department of Biosciences, Faculty of Science, Universiti Teknologi Malaysia, Skudai, Johor, Malaysia

**Keywords:** plant-microbe interaction, soil metagenome, soil microbiome, rhizocompartments, plant growth-promoting rhizobacteria (PGPR)

## Abstract

Soil nutrients and microbiota are known as essential components for healthy plant growth and crop productivity. However, limited studies have been conducted on the importance of soil microbiota in the early growth of oil palm seedlings (*Elaeis guineensis* Jacq.) under the influence of nitrogen, phosphorus and potassium (NPK) compound fertilizer (nitrogen, phosphorus, and potassium). In this study, we analyzed the root microbial community associated with seedlings grown under normal and sterilized soil conditions to ascertain the microbial strains potentially associated with soil, plant health and chemical fertilizer efficiency. Oil palm seedlings were grown under four treatments: (i) fertilized normal soil (+FN), (ii) unfertilized normal soil (−FN), (iii) fertilized sterilized soil (+FS) and (iv) unfertilized sterilized soil (−FS). Our findings revealed that chemical fertilizer promoted the growth of the copiotrophs Pseudomonadota and Bacteroidota in the control +FN, which are known to degrade complex polysaccharides. After autoclaving, the soil macronutrient content did not change, but soil sterilization reduced microbial diversity in the +FS and −FS treatments and altered the soil microbiota composition. Sterilized soil with a depleted microbial population adversely affected crop growth, which was exacerbated by fertilizer use. In the rhizosphere and rhizoplane compartments, a total of 412 and 868 amplicon sequence variances (ASVs) were found depleted in the +FS and −FS treatments, respectively. Several genera were identified in the ASVs with diminished abundance, including *Humibacter*, *Microbacterium*, *Mycobacterium*, 1921-2, HSB OF53-F07, *Mucilaginibacter*, *Bacillus*, *Paenibacillus*, and several unclassified genera, suggesting their possible roles in promoting the plant growth of oil palm seedlings. Soil sterilization might remove these beneficial microbes from the bulk soil pool, affecting the colonization ability in the rhizocompartments as well as their role in nutrient transformation. Therefore, this study provides useful insights concerning the benefits of a soil microbiome survey before making fertilizer recommendations.

## 1. Introduction

Oil palm is a perennial crop with a productive life of around 25 years. To maintain its growth and fruit production, oil palm requires a large amount of nutrients from the soil (Corley and Tinker, 2016). The depletion of soil nutrients over time fosters a dependency on chemical fertilizer. In Malaysia, the cost of fertilizer accounts for as high as 85% of the total production cost (Goh, 2005). To ensure the right balance of soil nutrients, an oil palm fertilizer recommendation system has been implemented as a general guideline (Goh, 2005). This system assists agronomists in resolving nutrient deficiencies in palm trees. However, pronounced differences have been found between the input amounts of nitrogen, phosphorus and potassium (NPK) applied in manuring and the output amount of NPK removed when the fruits were being harvested and carried away from the field (Tiemann et al., 2018). This unnecessary large insurance margin of fertilizer in oil palm can be attributed to the loss of nutrients due to leaching, inefficient nitrogen uptake by palms, poor nutrient retention in soil, nutrients’ antagonistic effect between fertilizers and other factors (Formaglio et al., 2020). In addition, multiple factors threaten the long-term sustainability of the oil palm industry, including ever-increasing fertilizer costs, the depletion of resources, labor shortages for multiple rounds of fertilizer applications and environmental concerns (Goh and Rolf, 2003; Woittiez et al., 2017).

Recent advancements in agriculture have led to the development of microbial-based biofertilisers that utilize plant growth-promoting rhizobacteria (PGPR) to promote overall plant growth and crop yield with a less adverse impact on the environment. A study on oil palm seedlings showed that microbial-based biofertilisers may improve plant nutrient uptake and soil nutrient retention, eventually reducing chemical fertilizer use (Ajeng et al., 2020). The area of PGPR living in the soil surrounding the plant roots is known as the rhizosphere; whereas the soil directly on the root surface is known as the rhizoplane (Carvalhais et al., 2013). The interaction of PGPR with plants and their effectiveness in plant growth promotion and yield productivity were reported in rice (Edwards et al., 2015; Zhang et al., 2019), wheat (Akitomo et al., 2016), grapevine (Cátia et al., 2014; Wu et al., 2020), soybean (Han et al., 2020) and sugar beet (Víctor et al., 2019). The biological roles of PGPR include nitrogen fixation, the solubilisation of mineral phosphate or potassium, an increase in host resistance against pathogens, an increase in the host’s ability to withstand biotic and abiotic stresses, and generating essential compounds or macromolecules such as siderophores, indoleacetic acid (IAA), enzyme 1-aminocyclopropane-1-carboxylate (ACC-deaminase), hormones and others (Yaish et al., 2016; Li et al., 2019; Rodriguez et al., 2019; Romano et al., 2020). The well-studied PGPR genera include *Pseudomonas*, *Azospirillum*, *Azotobacter*, *Bacillus*, *Burkholderia*, *Enterobacter*, *Rhizobium*, *Erwinia*, *Flavobacterium* and many more (Compant et al., 2019).

While plant–microbe interactions at the plant-soil interface have been shown to be crucial to nutrient acquisition and other benefits, little is known about how microbial interactions affect fertilizer uptake and nutrient exchange throughout the development of oil palm plants. Furthermore, palm growth is also influenced by biotic and abiotic factors (Woittiez et al., 2017; Goh et al., 2020), making it challenging to assess the sole effect of microbial activity associated with palm fruit yields. It was found that the sterilization of soil could improve plant growth performance by removing soil-borne pathogens from the soil, resulting in a random and natural recolonisation of beneficial microorganisms (Zhang et al., 2016; Li et al., 2019). However, the effects of sterilization on a “healthy soil microbiome,” microbial recovery and plant health has not been studied for oil palm. Thus, we designed an experiment to observe the effect of soil sterilization and NPK fertilization on the profile of microbial communities across bulk soil and rhizocompartments (the rhizosphere and rhizoplane) associated with the vegetative growth of oil palm seedlings. The microbial resilience theory proposed by Allison and Martiny (2008) states that changes in the microbial communities due to disturbances may directly affect ecosystem processes. Based on this concept, we hypothesized that oil palm seedlings may recruit a functional group of microbes from the surrounding soil onto their root-associated compartments to improve palm growth and development under soil sterilization and fertilization. By understanding the interactions between plants, microbes and chemical fertilizer, an effective biofertiliser can be developed to complement chemical fertilizer applications in the oil palm industry.

## 2. Materials and methods

### 2.1. Experimental soil

The soil used in this study was collected from an oil palm plantation field located at Banting Estate, Malaysia (2°47′3″N, 101°27′55″E). According to USDA (United States Department of Agriculture) soil taxonomy, the soil was classified as a loamy clay texture (26% sand, 31% silt, and 42% clay). After mixing, a portion of the collected soil samples was autoclaved thrice (at 121°C, 15 psi, 45 min for each cycle) to generate an initially pre-sterilized soil. The soil kept in the sealed bags was allowed to cool overnight between each set of autoclaving processes. Pre-sterilized soils were used in order to eliminate microbial communities indigenous to the soil. A complete elimination was confirmed by the absence of colonies on the nutrient agar after 48 h of incubation using spread plate technique.

### 2.2. Experimental design

A total of 80 germinated oil palm commercial seeds (*Deli Dura* × *AVROS Pisifera*) were obtained from the Oil Palm Breeding Unit, Sime Darby Plantation Research Sdn. Bhd., Malaysia. These full-sib seeds were derived from a fresh fruit bunch to achieve genetic uniformity. The seeds that qualify for sowing must have a radicle and plumule of approximately 1 cm in length. Each seed was sowed in a polybag (25 cm × 30 cm) filled with an equal weight of either normal or pre-sterilized soils. Seedlings were subjected to four treatments including (i) fertilized normal soil (+FN), (ii) unfertilized normal soil (−FN), (iii) fertilized sterilized soil (+FS), and (iv) unfertilized sterilized soil (−FS), in a complete randomized designed trial with 20 replicates per treatment. The abbreviations +F referred to the polybags applied with fertilizer, while −F referred to the fertilizer-free polybags, either using natural normal soil (N) or pre-sterilized soil (S). Under the normal commercial practice, oil palm seedlings were raised in the main nursery for 12 months, supplemented with compound fertilizer. Hence, the +FN control was served as a positive control to compare against other treatments in this study. The seedlings were raised in an open space nursery owned by Sime Darby Plantation near Banting. Each polybag was placed on top of a concrete slab to prevent the roots from penetrating the earth. After 6 weeks of sowing, the granular compound fertilizer N-P-K-Mg (14:13:9:2.5:trace elements, CCM Fertilizers Sdn. Bhd., Malaysia) was introduced into these seedlings according to the fertilizer schedule and dosage stated in the Sime Darby Plantation’s standard agriculture reference manual (unpublished). The complete fertilizer application throughout the 8 month trial was 100 g of granular NPK fertilizer per polybag. Upon the treatment effects were observed on these palms, a destructive sampling was conducted using 10 randomly selected seedlings from each treatment. Plant height, the number of developed fronds and total dry biomass were recorded accordingly.

### 2.3. Segregation of microbiota in bulk soil and rhizocompartments

In each treatment, five randomly selected seedlings were subjected to soil–root washing to segregate the rhizocompartment. Each oil palm seedling was carefully excavated from the polybag. Loose bulk soil from each seedling was collected and preserved individually at −80°C. The exposed root was vigorously shaken to remove the loosely adhering soil for collecting the rhizosphere microbiome. Soil bound firmly on the root was treated as rhizoplane. Rhizosphere and rhizoplane pellets from the same seedling were obtained by repeated chemical washings and centrifugations as described by Wieland et al. (2001). In total, we performed five replications across three compartments (bulk soil, the rhizosphere and rhizoplane) for +FN control, −FN, +FS, and −FS treatments.

### 2.4. DNA extraction and amplicon sequencing

Total genomic DNA extractions for each sample were performed using FastDNA™ Spin Kit for Soil (MP Biomedical, USA) following the manufacturer’s protocol. The quality and quantity of the extracted DNA were measured using agarose gel electrophoresis and Qubit spectrometer (Invitrogen, USA). The V3-V4 hypervariable regions of the prokaryotic 16S rRNA gene were amplified using primer 341F and 806R (Bates et al., 2011). Five replicates of all treatments except the +FN control in rhizoplane (only four replicates due to a problem with one sample) were sequenced individually using paired-end (2 bp × 250 bp) protocol on an Illumina HiSeq platform (Illumina, USA). This has accounted to a total of 59 samples.

### 2.5. Bioinformatics and statistical analyses

Paired-end reads merging, assigning, trimming, and quality filtering were performed using DADA2 pipeline v1.8, from an open source R package (Callahan et al., 2016). All the parameters set were by default. Primer-trimmed sequences with more than 200 bp without ambiguous base calls were quality filtered using the “filterAndTrim” function in the same pipeline. Subsequently, the estimated error rate was learned and calculated using the machine learning algorithm implemented in DADA2 from the subsampled reads. Forward and reverse sequences were merged to obtain amplicon sequence variant (ASV). Chimeric sequences were eliminated, resulting in a sequence length ranging from 400 to 450 nucleotides. Taxonomic classification of each ASV was annotated using SILVA reference database (SILVA 138 release) (Quast et al., 2013). After removing chloroplast and mitochondria, we further removed trace sequences of microbial read singleton, doubleton, and retained only ASVs with more than 10 reads in at least 60% of samples (*n* = 5), for each treatment independently. Sequencing data were rarefied to the lowest number of reads observed in a single sample, that is 23,124 reads.

Statistical analyses were conducted using R version 4.2.1 (R Core Team, 2020). To compare between treatments, significant differences in the soil, plant nutrient and alpha diversity were evaluated with non-parametric Kruskal–Wallis test and followed by *post hoc* comparisons conducted in pairwise Wilcoxon tests (*p*-value < 0.05). The alpha–diversity were represented by Chao1 richness (Chao, 1984) and Shannon index (Shannon, 1948). The treatment effect on the overall soil–root microbiome composition was assessed using permutational analysis of variance (PERMANOVA) with the “adonis2” function, and the treatment centroids was calculated using betadisper, both from the community ecology R “vegan” package (Oksanen et al., 2019). To visualize the microbial composition differences, unconstrained principal coordinate analysis (PCoA) was analyzed using the R package “phyloseq” (Paul and Susan, 2013) and plotted with “ggplot2” function (Wickham, 2016). We performed a differential abundance DESeq2 analysis (Love et al., 2014) to identify the ASVs that were significantly varied in rhizocompartments across treatments by comparing to +FN control. The significant value was reported at adjusted *p*-value < 0.01 with abundance in log2 fold change greater than 2. Heatmap of the relative abundance of archaea was generated by R “pheatmap” package (Kolde, 2019). The resulting sequencing data are publicly available at NCBI SRA under Bioproject PRJNA818730.

### 2.6. Physicochemical analyses of soil and plant samples

With the same subset of 59 samples, we performed soil and plant chemical analyses. Bulk soils were collected for physicochemical analysis. The soil samples were oven-dried at 70°C, ground and subsequently sieved to pass through a 5 mm sieve. These soil samples were then analyzed for pH, carbon/nitrogen ratio (C/N ratio), total nitrogen (TN), total phosphorus (TP), available phosphorus (AP), exchangeable cations included potassium (K), calcium (Ca), magnesium (Mg) and electrical conductivity (EC). The plant samples were also analyzed separately with TN, TP, K, Mg and Ca. Using an elemental analyser, soil and plant samples were analyzed for total C and N by the dry combustion method (Matejovic, 1993). Total phosphorus was determined by the vanadate-molybdate method (Tandon et al., 1968) using a spectrophotometer at 425 nm. Cations including K, Ca, and Mg were extracted using 1 M ammonium acetate (NH_4_OAc) calibrated at pH 7 followed by inductively coupled plasma (Perkin Elmer, USA) (David, 1960). The EC of the soil was measured by mixing soil and water at a 1:2 ratio (w/v), and then using a conductivity meter (Mettler Toledo, USA) to determine the EC of the resulting mixture (Heiniger et al., 2003). All the analyses were completed by Espek Research and Advisory Services (ERAS) laboratory, based on the M.S 678 (soil) method outlined by the Standard and Industrial Research Institute of Malaysia (Malaysian Standard [MS], 1980).

## 3. Results

### 3.1. Seedling growth responses to the presence of fertilizer in normal and pre-sterilized soils

**FIGURE 1:**
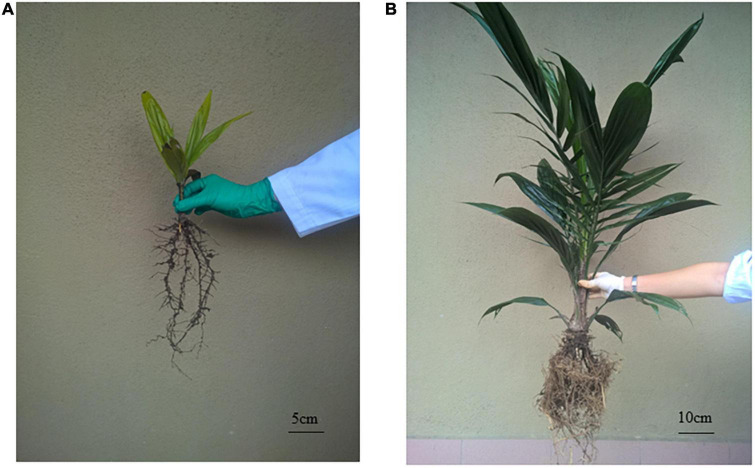
Oil palm seedling’s phenotype after 8 months planting for **(A)** +FS treatment and **(B)** +FN control. Replication of each treatment is in Supplementary Figure 1.

**FIGURE 2:**
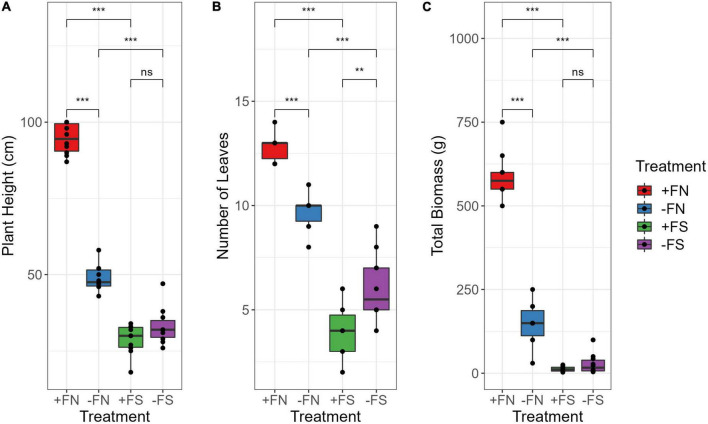
Fertilizer application in the normal soil significantly affect **(A)** plant height, **(B)** number of leaves, and **(C)** total dry biomass. Besides, sterilization significantly impacted the plants grown in +FS and –FS soil compared to +FN soil while no significant difference observed among the sterilized soil seedlings. Asterisk indicate significant differences among treatments using Kruskal–Wallis test and adjusted with *post hoc* pairwise Wilcoxon test, where ** denotes *p* < 0.01, ***denotes *p* < 0.001 and ns denotes non-significant. *n* = 10. +FN, fertilized normal soil; –FN, unfertilized normal soil; +FS, fertilized sterilized soil; –FS, unfertilized sterilized soil.

Seedlings grown in normal soils (+FN and −FN) appeared to be healthy after 8 months of planting; however, seedlings grown in pre-sterilized soils (+FS and −FS) were stunted despite fertilizer being applied to the +FS treatment (Figure 1 and Supplementary Figure 1). Distinctive plant height, the number of leaves and total biomass were observed for each treatment (Figures 2A–C). The control +FN seedlings were significantly taller and heavier, with a mean of 94.5 cm and 590 g, as compared with seedlings grown in normal unfertilized soil (−FN) with a mean of 48.9 cm and 148 g (*p* < 0.001). Seedlings in both +FN and −FN had an average leaf count of 13 and 10, respectively, with lanceolate, bifurcate and pinnae observed and recorded. In general, seedlings that were grown in the −FN treatment displayed slower growth compared to the +FN control. On the other hand, seedlings that were grown in the pre-sterilized soils (−FS and +FS) had stunted growth with a short plant height, remaining within the range of 18–33 cm, and an average total biomass of 20 g. Furthermore, the leaf development of these seedlings stopped at month five after sowing, without any pinnae observed.

### 3.2. Treatment effect on chemical properties in normal and pre-sterilized soils

In order to ensure autoclaving did not alter the soil chemistry, the pH and nutrient contents of the soil were measured before and after autoclaving. As shown in Table 1, the pH, macronutrient and micronutrient content in normal and pre-sterilized soils did not differ significantly (*p* > 0.05). After 8 months, the effect of fertilizer application on the soil nutrient content was remarkable, where TP, AP, K and Mg showed at least a two-fold increase with the treatment +FN and +FS treatments compared to the unfertilized counterpart soils of −FN and −FS (Table 1). Moreover, the chemical fertilizer raised the EC slightly to 75 μS/cm in the +FN control, whereas a sharp increase in the EC of 336 μS/cm took place in the +FS treatment.

**TABLE 1:** Effect of autoclaving on the initial soil properties and effect of treatments on soil properties after 8 months of planting.

Parameters	Normal soil		Pre-sterilized soil	
**Before treatment**				
pH	4.1 ± 0.1^a^		3.9 ± 0.4^a^	
Electrical conductivity (μS/cm)	76.67 ± 24.22^a^		70.0 ± 12.65^a^	
**Soil nutrient concentration**				
C/N ratio	34.16 ± 55.05^a^		11.67 ± 5.24^a^	
Total nitrogen (%)	0.19 ± 0.08^a^		0.22 ± 0.04^a^	
Total phosphorus (mg/L)	389.67 ± 111.85^a^		387 ± 48.54^a^	
Available phosphorus (mg/L)	43.33 ± 38.10^a^		48 ± 17.93^a^	
Potassium (cmol/kg)	0.49 ± 0.20^a^		0.41 ± 0.17^a^	
Magnesium (cmol/kg)	0.78 ± 0.31^a^		0.74 ± 0.25^a^	
Calcium (cmol/kg)	2.01 ± 0.64^a^		1.62 ± 0.45^a^	
**Parameters**	**+FN**	**−FN**	**+FS**	**−FS**
**Treatments**				
pH	4.4 ± 0.2^a^	4.2 ± 0.1^a,b^	4.0 ± 0.2^b^	4.2 ± 0.1^a,b^
Electrical conductivity (μS/cm)	75.0 ± 5.77^b^	57.5 ± 5.0^b^	336 ± 70.21^a^	57.5 ± 5.0^b^
**Soil nutrient concentration**				
C/N ratio	16.0 ± 9.03^a^	16.6 ± 10.31^a^	16.8 ± 3.9^a^	20.0 ± 5.0^a^
Total nitrogen (%)	0.25 ± 0.06^a^	0.24 ± 0.08^a^	0.26 ± 0.02^a^	0.27 ± 0.06^a^
Total phosphorus (mg/L)	930.8 ± 409.96^a^	328.00 ± 36.36^b^	845.0 ± 438.58^a,b^	290.6 ± 51.34^b^
Available phosphorus (mg/L)	146.6 ± 34.59^a^	74.6 ± 49.39^a^	208.4 ± 157.47^a^	75.6 ± 65.19^a^
Potassium (cmol/kg)	0.66 ± 0.15^b^	0.27 ± 0.02^d^	1.82 ± 0.21^a^	0.35 ± 0.05^c^
Magnesium (cmol/kg)	1.91 ± 0.47^b^	0.53 ± 0.05^c^	3.49 ± 0.91^a^	0.63 ± 0.15^c^
Calcium (cmol/kg)	2.90 ± 0.42^a^	2.49 ± 0.34^a,b^	2.63 ± 0.47^a,b^	2.06 ± 0.28^b^

^a,b,c,d^Indicate significant differences according to Kruskal–Wallis test (p < 0.05). Values are presented as mean value ± SDs (n = 5).+FN, fertilized normal soil; −FN, unfertilized normal soil; +FS, fertilized sterilized soil; −FS, unfertilized sterilized soil.

### 3.3. Treatment effect on plant nutrient content in normal and pre-sterilized soils

The effect of the fertilizer treatment on the plant nutrient content was examined after 8 months of planting (Table 2). As a result of the low plant biomass obtained with the pre-sterilized soil treatment, plant nutrient analysis was not possible for the +FS and −FS treatments. Therefore, only the +FN and −FN treatments were analyzed in this study. The TN content was not significantly different between these two treatments; however, the parameters of TP, K, Mg and Ca content were significantly lower in the −FN treatment.

**TABLE 2:** Effect of different treatments on the plant nutrient concentration after 8 months of planting.

Treatments				
Parameter	+FN	−FN	+FS	−FS
**Plant nutrients concentration**				
Total nitrogen (%)	2.53 ± 0.14^a^	2.70 ± 0.07^a^	na	na
Total phosphorus (%)	0.36 ± 0.10^a^	0.21 ± 0.03^b^	na	na
Potassium (%)	2.88 ± 0.65^a^	2.11 ± 0.12^b^	na	na
Magnesium (%)	0.42 ± 0.08^a^	0.31 ± 0.02^b^	na	na
Calcium (%)	0.59 ± 0.10^a^	0.43 ± 0.05^b^	na	na

^a,b^Indicate significant differences according to Kruskal–Wallis test (p < 0.05). Values are presented as mean value ± SDs (n = 5). na, data not available.

### 3.4. Effect of fertilizer and sterilization on root-soil-microbiota diversity

#### 3.4.1. Alpha and beta diversity

**FIGURE 3:**
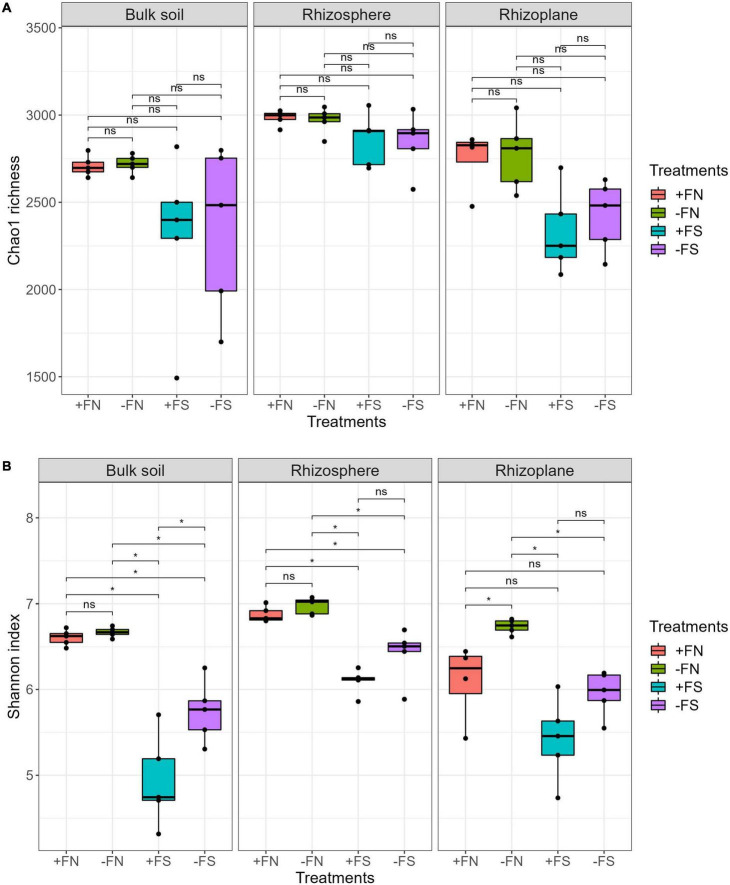
**(A)** Chao1 microbial richness were not affected by soil sterilization nor fertilizer. **(B)** Shannon index indicates the reduction of the microbial diversity in pre-sterilized soil system (+FS, –FS). Asterisk (*) indicate significant differences among treatments using Kruskal–Wallis test and adjusted with *post hoc* pairwise Wilcoxon test, with *denotes padj < 0.05 and ns denotes non-significant. *n* = 5.

We used the Chao1 index to measure species richness and the Shannon index to measure the diversity of the species in our study. Statistical testing showed no difference was found with the Chao1 richness estimator for the treatments and the +FN control (Figure 3A). Overall, the rhizosphere had the highest richness (Chao1: 2,715–3,055) followed by the rhizoplane (2,085–3,041) and bulk soil (1,492–2,818). The Chao1 index estimates missing taxa in a community, giving more weight to low-abundance taxa, and measures species richness. The Shannon index was used to explore the prokaryotic diversity of the oil palm seedlings, which showed the difference between the control and treatments within three main compartments. In each compartment, the Shannon index for the +FN control was slightly lower than that of the −FN treatment, but this difference is not statistically significant except for in the rhizoplane (Figure 3B). On the other hand, lower diversity (Shannon) was found in the bulk soil, rhizosphere and rhizoplane of the +FS and −FS treatments compared to the +FN control, which indicates that a reduction in microbiota diversity happened across all compartments in pre-sterilized soils. Lower microbial diversity in the soil–root compartments reflects an uneven prokaryotic population in the pre-sterilized soil.

**FIGURE 4:**
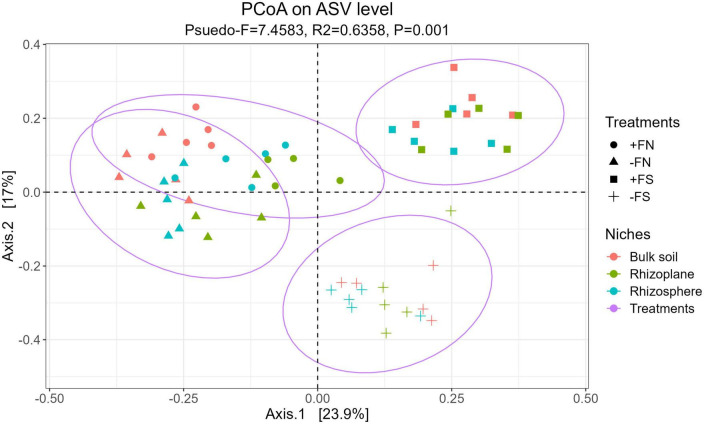
Overall model of unconstrained PCoA with Bray–Curtis distance showed that the soil microbiota were clustered accordingly to treatments (*P* < 0.001, PERMANOVA by adonis2).

Unconstrained principle coordinate analysis based on the Bray–Curtis distance metric was performed to test for dissimilarities in the microbial composition among treatments. Using PERMANOVA, it was demonstrated that there was a clear, significant separation between the microbiota of the treated and +FN control samples, as shown in Figure 4 (*p*-value < 0.001, *R*^2^ = 0.6358 and Pseudo-F = 7.45). Seedlings from the −FS and +FS treatments were clearly isolated and shifted to the right, and this treatment effect was confirmed with a significant result in the PERMANOVA (*p*-value < 0.001, *R*^2^ = 0.4000 and Pseudo-F = 17.20). Furthermore, the statistical test indicated that compartments had distinct microbiota compositions (*p*-values < 0.001, *R*^2^ = 0.1230 and Pseudo-F = 7.93), and the interaction between the treatments and compartments also showed a significant difference (*p*-values < 0.001, *R*^2^ = 0.1128 and Pseudo-F = 2.52). The homogeneity of group dispersions (PERMDISP) was significant (Pseudo-F = 3.6284 and *p*-value < 0.05), indicating that the differences observed in the model were at least partially driven by within-group ASV variation.

#### 3.4.2. Taxonomic microbial profile changes

**FIGURE 5:**
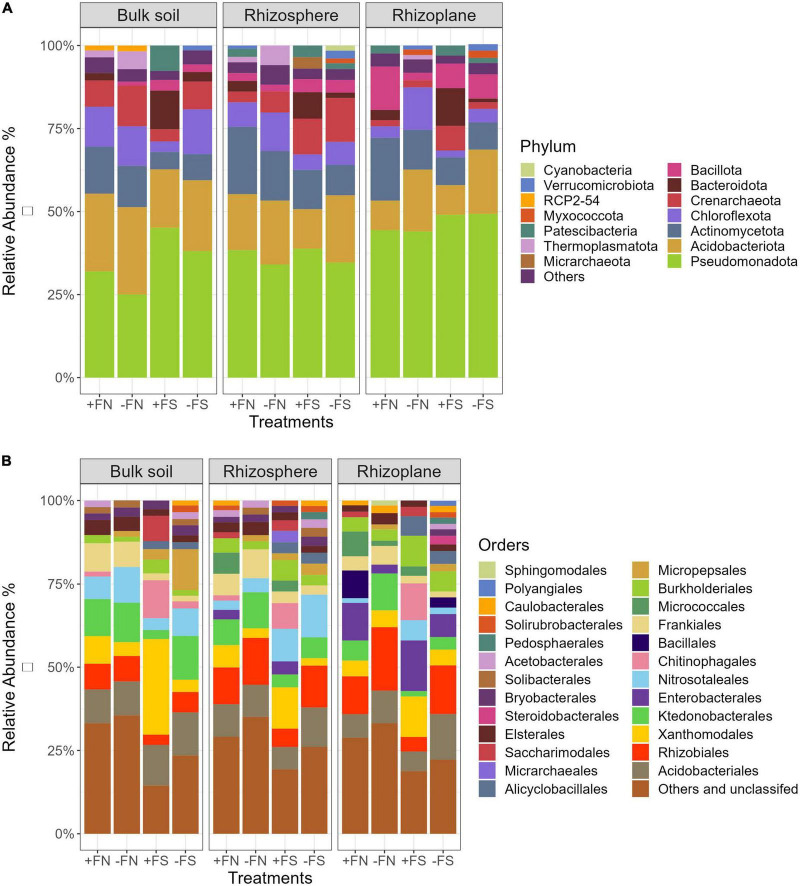
Average relative abundance of prokaryotic phyla and orders that are more than 1% abundance. Replication *n* = 5 for each niche and soil treatment. **(A)**–phylum level; **(B)**–order level.

The results generated 3,393 prokaryotic ASVs, as well as a total of 2,043,191 high-quality sequence reads with an average of 34,630 reads per sample. These ASVs were assigned to 27 phyla, 53 classes, 109 orders, 152 families and 255 genera. Figure 5A shows that there were five dominant bacterial phyla (Pseudomonadota, Acidobacteriota, Actinomycetota, Chloroflexota, and Bacteroidota) and one archaeal phylum (Crenarchaeota) found in the bulk soil, rhizosphere and rhizoplane compartments, accounting for > 80% of the prokaryotic profile. In the bulk soil and rhizosphere, bacterial communities exhibited similar taxonomic compositions, whereas they exhibited slight variations in the rhizoplane. Specifically, Crenarchaeota were present in both the bulk soil and rhizosphere, but only an average of 4% was found in the rhizoplane. The result also showed that the abundance of Bacillota increased twofold when closer to the oil palm root rhizoplane in all treatments.

A breakdown of microbial communities at the order level mainly included Acidobacteriales, Rhizobiales, Xanthomonadales, Ktedonobacteriales, Nitrosotaleales, and Frankiales, accounting for 32–52% of the total number of bacterial communities (Figure 5B). Simultaneously, the number of unclassified bacteria found in the various compartments in this study was as high as 35%. Like the phyla level, the bulk soil and rhizosphere demonstrated a similar order composition, with the rhizoplane exhibiting slight variations. For example, the rhizoplane compartment showed increases in Enterobacteriales and Burkholderiales (phylum Pseudomonadota), as well as Bacillales (phylum Bacillota), and decreases in Nitrosotaleales (phylum Crenarchaeota) and Bryobacterales (phylum Acidobacteriota) across treatments compared with the bulk soil and rhizosphere.

The treatment effect of pre-sterilized soils (+FS and −FS) on the microbial composition was significant in the three different compartments of bulk soil, rhizosphere and rhizoplane. Compared to the +FN control, the relative abundances of Chitinophagales (phylum Bacteroidota), Micropepsales (phylum Pseudomonadota), Burkholderiales (phylum Pseudomonadota) and Alicyclobacillales (phylum Bacillota) were significantly increased, but those of the Ktedonobacteriales (phylum Chloroflexota), Rhizobiales (phylum Pseudomonadota) and Frankiales (phylum Actinomycetota) were significantly decreased in the three compartments.

The chemical fertilizer impacted the soil–root microbial communities to a certain extent. The application of fertilizer in both normal and sterilized soil significantly decreased the relative abundances of Nitrosotaleales (phylum Crenarchaeota), Ktedonobateriales (phylum Chloroflexota) and increased those of Xanthomonadales (phylum Pseudomonadota), Burkholderiales (phylum Pseudomonadota), Chitinophagales (phylum Bacteroidota) and Micrarchaeales (phylum Micrarchaeota). Specifically, soil sterilization and NPK fertilizer application had an interactive effect on the relative abundances of Xanthomonadales, Enterobacteriales, Chitinophagales and Burkholderiales in the three compartments.

#### 3.4.3. Enrichment effect of specific taxa among treatments

**FIGURE 6:**
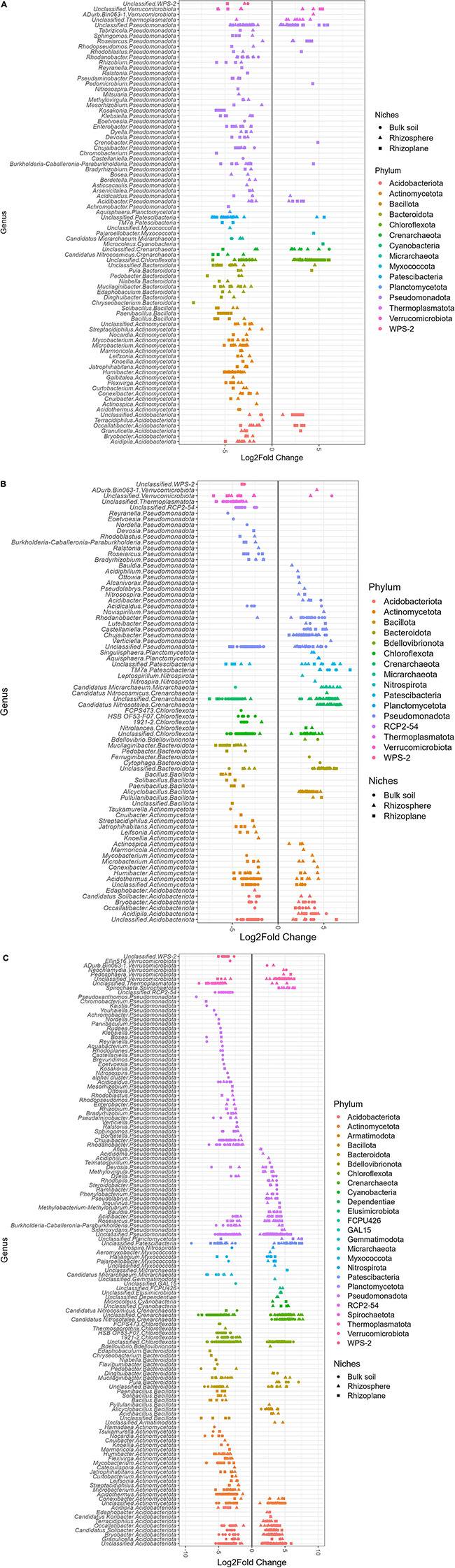
Differential abundance plots showing the significant log2 fold changes of amplicon sequence variants (ASVs) for **(A)** –FN, **(B)** +FS, and **(C)** –FS relative to control +FN. ASV taxonomic assignments were performed at the genus level whenever possible with respective phyla named behind it. Log2 fold change < 0 is depleted ASVs relative to +FN; log2 fold change > 0 is enriched ASVs relative to +FN. Assessed using DESeq2 at significant padj < 0.01.

Even though bacterial communities in the rhizocompartments originate from the bulk soil, our results indicated that the treatment effect introduced to the soil earlier caused varying degrees of significant differences in the microbial community structure. As shown in Table 3, different treatments significantly enriched or significantly depleted ASVs in comparison with the +FN control. Compared to the microbial communities of the +FN control, those of −FN/+FS/−FS soil recorded a total of 123/372/677 significantly enriched ASVs, and 372/412/868 significantly depleted ASVs, respectively. The −FN treatment showed a smaller difference in bacterial community structure and composition than the +FN control since fertilizer was the only factor involved. Out of the total 123 enriched ASVs, the bulk soil, rhizosphere and rhizoplane accounted for 2, 53 and 68 enriched ASVs in the −FN treatment relative to the +FN control (Supplementary Table 1). Among the 372 significant depleted ASVs in the −FN treatment, the rhizosphere contributed the highest number of depleted ASVs (175), followed by the rhizoplane (166) and bulk soil (31) (Supplementary Table 4). Based on the enrichment–depletion plot, the −FN treatment showed multiple depletions of ASVs coming from the phyla of Acidobacteriota, Actinomycetota, Bacteroidota, Bacillota, Patescibacteria, and Pseudomonadota; meanwhile, multiple enrichments of ASVs were mainly from unclassified genus in the phyla of Chloroflexota and Pseudomonadota compared to the +FN control (Figure 6A). In addition, there was a substantial enrichment of archaeal ASVs from Thermoplasmatota and Crenarchaeota in the rhizosphere.

**TABLE 3:** Enriched and depleted ASVs in relative to +FN control.

Enriched ASV		Treatments		
**Compartments**	**Compared to control**	**−FN**	**+FS**	**−FS**
Bulk soil	+FN	2	121	175
Rhizosphere	+FN	53	197	333
Rhizoplane	+FN	68	54	169
Total		123	372	677
		Supplementary Table 1	Supplementary Table 2	Supplementary Table 3
**Depleted ASV**		**Treatments**		
**Compartments**	**Compared to control**	**−FS**	**+FS**	**−FS**
Bulk soil	+FN	31	283	599
Rhizosphere	+FN	175	48	134
Rhizoplane	+FN	166	81	135
Total		372	412	868
		Supplementary Table 4	Supplementary Table 5	Supplementary Table 6

Differential abundance analysis was performed with DESeq2 at significant padj < 0.01. Values were presented as number of ASVs being enriched and depleted in relative to +FN control. Enriched ASVs was depicted as fold change > 0, while depleted ASVs was depicted as fold change < 0. Details of the ASVs may refer to the Supplementary Tables 1–6.

Compared to the +FN control, pre-sterilized soils +FS and −FS resulted in greater differences in the bacterial community structure (Table 3). The application of chemical fertilizer in the −FS treatments enhanced the enrichment of ASVs in pre-sterilized bulk soils by 126, in the rhizosphere by 197 and in the rhizoplane by 54 (Supplementary Table 2); however, it depleted the number of ASVs in the pre-sterilized bulk soil by 283, in the rhizosphere by 48 and in the rhizoplane by 81 (Supplementary Table 5). Several unclassified genera from the phyla Acidobacteriota, Chloroflexota, Pseudomonadota, Crenarchaeota and Thermoplasmatota were depleted in the +FS bulk soil; meanwhile, *Chujaibacter*, *Castellaniella*, and *Rhodanobater* from the class Gammaproteobacteria were significantly enriched in the rhizocompartment (Figure 6B). It was found that the −FS treatment exhibited the highest enrichment and depletion of ASVs among treatments, with 176 enriched in the bulk soil, 333 enriched in the rhizosphere and 169 enriched in the rhizoplane (Supplementary Table 3); meanwhile, 599 were depleted in the bulk soil, 134 were depleted in the rhizosphere, and 135 were depleted in the rhizoplane (Supplementary Table 6). From the enrichment–depletion plot in Figure 6C, several unclassified genera from the phyla Acidobacteriota, Crenarchaeota, Chloroflexota and Pseudomonadota were depleted in the −FS bulk soil, and a few unclassified genera from the phylum Pseudomonadota were enriched in the rhizocompartment. From Figures 6B, C, it can be seen that both +FS and −FS treatments exhibited similar depletion pattern in the genera in the rhizocompartment, which were *Acidothermus*, *Humibacter*, *Microbacterium*, *Mycobacterium* (phylum Actinomycetota), 1921-2, HSB OF53-F07 (phylum Chloroflexota), *Mucilaginibacter* (phylum Bacteroidota), *Bacillus*, *Paenibacillus* (phylum Bacillota) and a few unclassified genera from the phyla Crenararchaeoata, Thermoplasmatota and RCP2-54.

**FIGURE 7:**
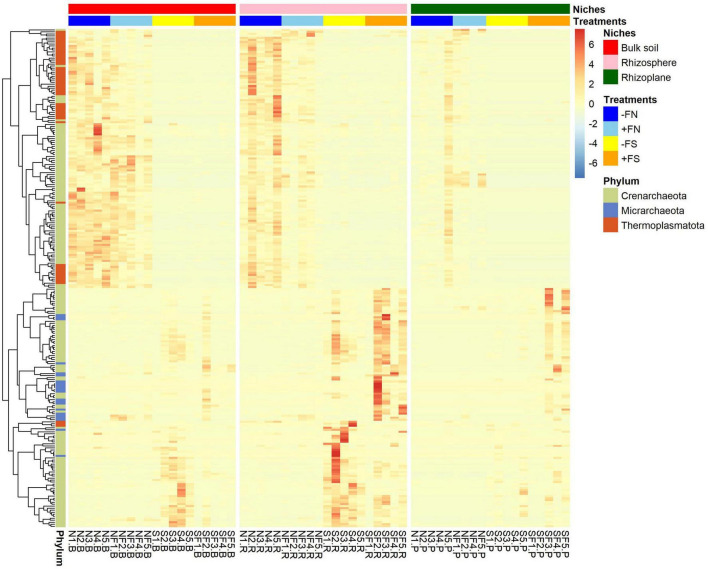
Heatmap of the relative abundances of archaeal ASVs. Vertical columns show treatments found in each compartment and horizontal rows represent ASVs. Rows are clustered in accordance with the relative abundance within that treatment. Z-score with the scale bar shows the gradient of color saturation representing the relative abundance of the organisms.

An additional analysis of the archaeal community was performed by plotting a heatmap of the relative abundance of archaea (Figure 7). According to the heatmap analysis, the normal soil samples clustered differently compared to the pre-sterilized soil samples. It was found that Crenarchaeota- and Thermoplasmatota-affiliated ASVs were most prevalent in normal soil (−FN) and normal fertilized soil (+FN), while Micrarchaeota-affiliated ASVs were most prevalent in pre-sterilized fertilized soil (+FS) and *Candidatus Nitrosotalea* (phylum Crenarchaeota) was most prevalent in pre-sterilized soil (−FS). This is in accordance with our analysis of differential abundances.

## 4. Discussion

### 4.1. Effect of soil sterilization and fertilization on microbiota and growth of seedlings

In this study, both +FS- and −FS-treated oil palm seedlings without pinnae suffered severely, whereas −FN seedlings grew slower than +FN control seedlings. Interestingly, despite similar environmental conditions and the same amount of NPK granules being supplied, palm growth in +FS was markedly different from that in +FN. Our results showed that the autoclave did not significantly change the soil macronutrient content. However, a few studies suggested that heat sterilization increased soil nutrient availability (Mahmood et al., 2014; Oruru et al., 2018; Li et al., 2019). As reported in the oil palm soil fertility classification guidelines by Goh and Rolf (2003), the soil macronutrients (NPK concentration) applied in our study were at the optimum level for oil palm growth before and after 8 months of planting. Therefore, the nutrient availability of N, P and K was not the limiting factor causing stunted growth in both +FS and −FS treatments. We observed a high accumulation of soil nutrient content such as K, Ca and Mg cations, resulting from the release of compound fertilizer in the +FS treatment relative to the +FN control. This was evidenced by the two-fold increase in the soil EC value in the +FS treatment. A high EC value can affect plant nutrient availability and soil microorganism activity. A previous study showed that osmotic pressure primarily caused by excess K, Mg, Ca, Na and Cl ions, may affect microbial activity or even suppress the growth of certain microbes by drying or lysing their cells (Yan et al., 2015). Although the sterilization process eliminated indigenous microbes from the soil, living microbes re-established themselves in polybags during seedling cultivation in the pre-sterilized soil. However, the microbial diversity in the +FS treatment remained low compared to the +FN control. The acquisition of this new microbial community in pre-sterilized soil was a random natural incidence which may result from irrigation, rain, human activity or insect activity. The results suggest that the stunted palm growth in the +FS treatment could be due to this excessive accumulation of soluble salts in the root zone that hindered the plant roots from withdrawing water from the surrounding soil (Sogoni et al., 2021), thus limiting plant nutrient uptake and affecting the microbial assemblage around the root zone at the end of the planting.

Salts and nutrients released from compound fertilizer were readily absorbed by the plant, as evidenced by the high amount of total dry biomass and optimal soil K, Ca and Mg cations in the +FN control. Although compound fertilizer had a significant impact on plant growth, PCoA and taxonomy plots revealed that the microbiota in the normal soil +FN control and −FN treatment differed slightly. This is similar to the results previously reported in woodland soil (Karki et al., 2021), where a low pH and low nutrient status in −FN favored the growth of oligotrophs such as Acidobacteriota, whereas fertilizer inputs increased the relative abundance of copiotroph in the +FN control, such as Bacteroidota and Pseudomonadota (formerly known as Proteobacteria), which prefer nutrient-rich conditions for growth. Copiotrophic bacteria are specialized in degrading complex polysaccharides (Spring et al., 2015). Nakayama et al. (2021) reported that microbial species of Pseudomonadota and Bacteroidota that shared a similar niche in forest soil could perform net nitrogen nitrification and mineralisation. Similarly, the phosphorus (P) applied via fertilization was quickly fixed into a stable form in the soil, requiring the help of phosphate solubilising bacteria to release the fixed P into a plant usable form. In this study, the +FN control was found to have high abundances of bacteria from the genera *Rhizobium* and *Enterobacter*, which may function as potential P solubilisers. Altogether, this suggests that the fertilizer provision in the +FN control altered the function of indigenous soil microbes toward improved nutrient acquisition or nutrient transformation for better plant growth. However, further validation is needed by checking the function of these native soil microbes.

### 4.2. Disrupted bulk soil microbiome led to imbalance in rhizospheric colonization

By the end of the study, microorganisms had built up in the pre-sterilized soil. Despite this, PCoA and PERMANOVA tests revealed a newly established soil microbiota in the −FS and +FS treatments that were distinct from the original indigenous microbiota found in −FN and +FN. Soil sterilization, regardless of the presence of fertilizer, also caused a reduction in microbial diversity across the bulk soil and rhizocompartments. Pre-sterilization may have reduced the number of native soil microbes, which may have influenced the stunted seedling growth observed in the palms under the −FS and +FS treatments. Several studies have shown that soil sterilization can eliminate soil-borne pathogens and promote changes in soil microbe communities that lead to improved plant growth and increased beneficial microbes (Mahmood et al., 2014; Zhang et al., 2016; Li et al., 2019). However, in our experiment, the soil used did not contain *Ganoderma boninense*, a fungal pathogen that infects oil palm trees in South East Asia (Jeffery Daim et al., 2015) (ITS data not shown). The benefits of soil sterilization, specifically the elimination of soil-borne pathogens, were therefore not applicable to this experiment.

**FIGURE 8:**
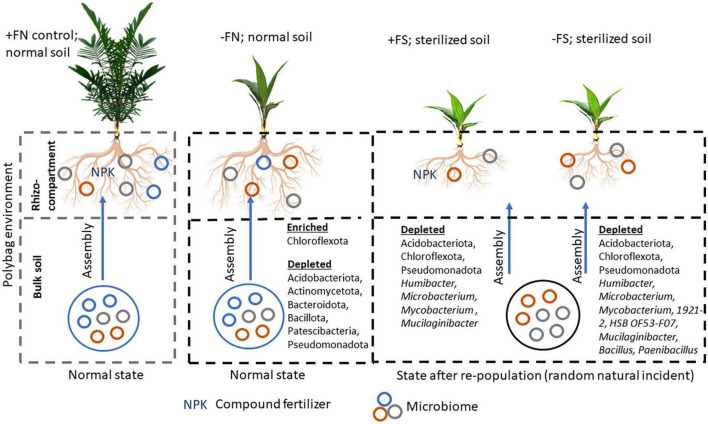
Plausible mechanism of bulk soil microbial community changes that lead to imbalance rhizospheric assemblage and plant fitness. Oil palm seedlings grow as vigorous and healthy when fertilizer is supplemented to the seedlings in normal soil. After soil sterilization, the bulk soil microbial community is altered. The acquisition of this new microbial community in pre-sterilized soil was a random natural incidence which may result from irrigation, rain, human activity, or insect activity.

A study by Oruru et al. (2018) on cowpeas found that plants grown in non-sterilized soil performed better than those that were grown in pre-sterilized soil. The findings suggest that early root colonization of mycorrhizae or beneficial microbes from the bulk soil is critical for maintaining plant and soil health. Therefore, we speculate that the pre-sterilized soil in our experiment may have eliminated functional microbes that were necessary for colonizing oil palm seedlings’ roots during the early stages of growth and development (Figure 8). This is evidenced by a high number of depleted bulk soil ASVs in the −FS (599) and +FS (283) treatments compared to the −FN treatment (31) (Table 3). Despite the fact that plant roots can regulate the diversity and abundance of the rhizospheric soil microbiome via root exudate secretion, the depletion of microbial sources in the pre-sterilized bulk soil system can disrupt the microbial network and cause some degree of stress to the plant. As a result, the plant may not be able to maintain its health through rhizosphere recruitment. This finding has also been reported in various crop studies where changes in the composition of the bulk soil microbiome significantly affected the root-associated rhizosphere and rhizoplane microbial communities (Edwards et al., 2015; Liu et al., 2019; Zhang et al., 2019; Han et al., 2020).

A significant number of enriched ASVs were found in the soil–root compartments of both the −FS and +FS treatments (Table 3). Among the enriched ASVs were *Rhodanobacter*, *Roseiarcus*, *Chujaibacter*, *Acidibacter*, *Alicyclobacillus*, *Bryobacter*, and *Occallatibacter*. None of these are pathogenic toward plants. The enrichment of these species may be attributed to the “priority effect,” which occurs when an early-arriving microbial community competes for space and resources at the plant roots, thereby preventing the establishment of a late-arriving community (Debray et al., 2022). Similarly, the −FN treatment did not detect any of the plant pathogenic bacteria that cause disease in economically important crops (Mansfield et al., 2012), including *Pseudomonas syringae* pathovars, *Ralstonia solanacearum*, *Agrobacterium tumefaciens*, *Xanthomonas oryzae* pv. *oryzae*, and *Erwinia amylovora*.

In addition to the bacterial community, soil archaeal communities were significantly affected in all of the treatments. Crenarchaeota, and Thermoplasmatota were the predominant archaeal phyla in the −FN and +FN treatments. These phyla are associated with nitrogen cycling (Nitrosopumilaceae, Nitrososphaeraceae, and Nitrosotaleaceae) (Wan et al., 2021), and carbon metabolism (Marine Group I) (Swan et al., 2014). Intriguingly, a noticeable reduction in Nitrosotaleaceae (*Candidatus Nitrosotalea*) and Nitrososphaeraceae was observed in pre-sterilized rhizosphere soil. This may be due to competitive interactions with bacterial nitrogen nitrifiers, particularly since *Nitrospira* abundance was approximately four times higher in the +FS and −FS treatments. While some clades in archaeal Crenarchaeota contribute significantly to nitrification and carbon cycling, we cannot conclude that enriched or depleted archaeal-associated ASVs are exclusively related to the health of oil palm seedlings.

## 5. Conclusion

In conclusion, this study profiled changes to the microbial community of oil palm seedlings grown in normal and pre-sterilized soils, and how these soils interacted with compound NPK fertilizer. Collectively, our results show that the NPK compound fertilizer enhanced the growth of plants in normal soil (+FN), and at the same time, enriched the copiotrophic bacteria Pseudomonadota and Bacteroidota to degrade complex polysaccharides. Microbiota diversity was significantly reduced in the +FS and −FS treatments compared to the +FN control. Furthermore, soil sterilization and NPK fertilizer application had an interactive effect which negatively affected the growth and microbiome composition of the oil palm seedlings by depleting the ASVs belonging to *Humibacter*, *Microbacterium*, *Mycobacterium*, 1921-2, HSB OF53-F07, *Mucilaginibacter*, *Bacillus*, *Paenibacillus*, and several unclassified genera. While oil palm seedlings can recruit microbiota into the root-associated compartment, soil sterilization disrupts the bulk soil microbiota balance by removing beneficial microbes. This loss of ASVs in the rhizocompartment could have a significant effect on plant fitness and soil microbiome stability. A further study is recommended to unveil the function of microbes associated with chemical fertilizer under normal soil conditions and the mechanisms of microbe–microbe interactions in influencing soil quality and plant growth.

## Data availability statement

The datasets presented in this study can be found in online repositories. The names of the repository/repositories and accession number(s) can be found below: https://www.ncbi.nlm.nih.gov/bioproject/PRJNA818730.

## Author contributions

LH conceived and designed the research. JD, LH, and JI performed the experiments. JI and CT managed the project and resources. JD performed DNA extraction, analyzed sequencing data, and data visualization. JD, LH, and KG wrote the manuscript. All authors contributed to the article and approved the submitted version.
