# Supplementary material for: Impact of sterilization and chemical fertilizer on the microbiota of oil palm seedlings
Source: Front Microbiol. 2023 Apr 27;14:1091755. doi: 10.3389/fmicb.2023.1091755 (PMC10172575; doi:10.3389/fmicb.2023.1091755)
Supplement: Supplementary file 1 [file Data_Sheet_1.pdf]

Supplementary Material

Impact of Sterilization and Chemical Fertilizer on the Microbiota of Oil Palm Seedlings

Joyce Yoon Mei Ding[1*], Li Sim Ho[1], Julia Ibrahim[1], Chee Keng Teh[1], and Kian Mau Goh[2*]

*** Correspondence:** Joyce Yoon Mei Ding and Kian Mau Goh
joyce.ding.yoonmei@simedarbyplantation.com and gohkianmau@utm.my

1 Supplementary Figures

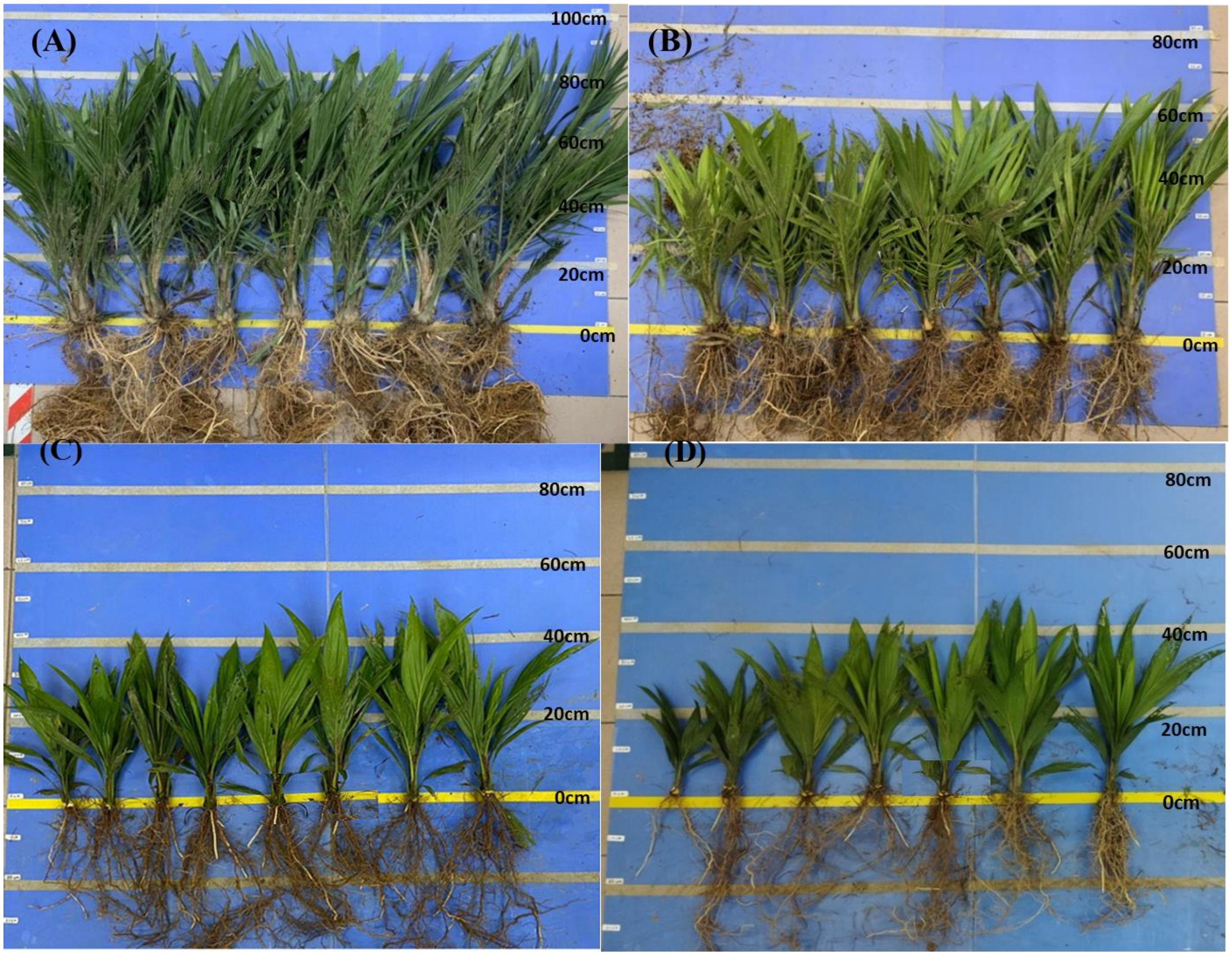

Supplementary Figure 1. Oil palm seedling's phenotype after 8 months planting for **(A)** +FN control, **(B)** Treatment −FN, **(C)** +FS, and **(D)** −FS. The yellow tape line indicates ground level at 0cm, each white marking tape line is an increase of 20cm in aboveground height. +FN, fertilized normal soil; −FN, unfertilized normal soil; +FS, fertilized sterilized soil; −FS, unfertilized sterilized soil.
